# Cognitive Impairment and Dementia: Gaining Insight through Circadian Clock Gene Pathways

**DOI:** 10.3390/biom11071002

**Published:** 2021-07-09

**Authors:** Kenneth Maiese

**Affiliations:** Cellular and Molecular Signaling, New York, NY 10022, USA; wntin75@yahoo.com

**Keywords:** Alzheimer’s disease, autophagy, circadian rhythm, dementia, erythropoietin, forkhead, FoxO, glymphatic pathway, mechanistic target of rapamycin (mTOR), Parkinson’s disease, silent mating type information regulation 2 homolog 1, sleep fragmentation

## Abstract

Neurodegenerative disorders affect fifteen percent of the world’s population and pose a significant financial burden to all nations. Cognitive impairment is the seventh leading cause of death throughout the globe. Given the enormous challenges to treat cognitive disorders, such as Alzheimer’s disease, and the inability to markedly limit disease progression, circadian clock gene pathways offer an exciting strategy to address cognitive loss. Alterations in circadian clock genes can result in age-related motor deficits, affect treatment regimens with neurodegenerative disorders, and lead to the onset and progression of dementia. Interestingly, circadian pathways hold an intricate relationship with autophagy, the mechanistic target of rapamycin (mTOR), the silent mating type information regulation 2 homolog 1 *(Saccharomyces cerevisiae*) (SIRT1), mammalian forkhead transcription factors (FoxOs), and the trophic factor erythropoietin. Autophagy induction is necessary to maintain circadian rhythm homeostasis and limit cortical neurodegenerative disease, but requires a fine balance in biological activity to foster proper circadian clock gene regulation that is intimately dependent upon mTOR, SIRT1, FoxOs, and growth factor expression. Circadian rhythm mechanisms offer innovative prospects for the development of new avenues to comprehend the underlying mechanisms of cognitive loss and forge ahead with new therapeutics for dementia that can offer effective clinical treatments.

## 1. The Significant Impact of Cognitive Loss and Neurodegenerative Disease

Non-communicable diseases (NCDs) affect a significant portion of the world’s population and it is believed that approximately 70 percent of the annual deaths that occur each year are the result of NCDs [1,2,3]. NCDs impact a large segment of the population in low- and middle-income countries. At least one-third of this population are under the age of 60 as compared to wealthier nations with only 10 percent of the population affected are under the age of 60 [1]. As an important component of NCDs, neurodegenerative disorders also lead to death and disability in a large proportion of the world’s population [4,5,6,7,8]. This is reflected in the role neurodegenerative diseases play in the ten leading causes of death that include cardiac disease, cancer, trauma, respiratory disease, stroke, Alzheimer’s disease (AD), diabetes, influenza and pneumonia, kidney disease, and suicide [9]. Neurodegenerative disorders include more than 600 disease entities and lead to disorders in almost one billion individuals throughout the globe [10,11,12,13,14,15]. This is equivalent to neurodegenerative diseases affecting 15 percent of the world’s population and leading to the death of at least 7 million individuals each year [8,16].

It is of interest to note that the age of the global population has been increasing with life expectancy approaching 80 years of age [17] and that the number of individuals over the age of 65 has doubled during the prior 50 years [6,14,18,19,20]. This includes developed nations, such as the United States (US), where life expectancy was decreasing over a four-year decline, but with a recent reduction in deaths from opioid overdoses, life expectancy has been increasing again [9]. Yet, as a result of this progressive increase in lifespan and improvements in global healthcare, it is predicted that neurodegenerative diseases will increase in prevalence (Table 1). As an example, dementia is now considered to be the 7th leading cause of death and dementia affects all countries throughout the world at a significant financial burden [2,13,14,21,22,23]. Almost 5 percent of the world’s elderly population, estimated at 50 million individuals, suffer from dementia. By the year 2030, 82 million people are expected to have dementia, and this will reach 152 million individuals by the year 2050.

Cognitive disorders affect more than 5 million individuals in the US alone [24] and almost 60 percent of dementia cases are the result from AD [8,24,25,26,27,28,29]. Interestingly, it is also believed that dementia is under diagnosed [30,31]. Although AD can affect a significant number of individuals in the world, familial presentations of AD only account for less than 2% of all cases [24]. In these cases, presenilin 1 or 2 gene mutations and amyloid precursor protein (APP) gene mutations can affect 200 families in the world and onset of the disease is before reaching age 55 [15,32,33]. In contrast, sporadic AD occurs at a later age, the ε4 allele of the apolipoprotein E (APOE) gene leads to increased risk, and the disease represents most cases for AD by affecting 10 percent of the population in the world.

In addition to the increasing prevalence of cognitive disorders, significant financial concerns also exist for dementia. Greater than 800 billion United States dollars (USD) is spent to care for individuals with dementia on an annual basis equaling almost 2 percent of the global gross domestic product. It has been predicted that by the year 2030, medical and social services could cost 2 trillion USD annually in the US. At present, more than 5 million individuals are diagnosed with AD and almost 4 million are under treatment at an annual cost of 3.8 billion USD. The annual market size for therapeutic treatments for AD may be underestimated and is expected to currently exceed 11 billion USD. At least 60 million new health and social care workers will be required to fill this need [1,2,34]. These projections do not take into account when to address the need for these healthcare workers in a timely manner. Cognitive loss and dementia may not be recognized until the late or end stages of the disease. This leaves limited time for effective treatment and may involve fragmented care.

## 2. Novel Therapeutic Considerations for Cognitive Loss

In general, most neurodegenerative disorders present significant hurdles during diagnosis, treatment, and ability to limit disease progression. Cognitive disorders, such as dementia with AD, raise this bar even further since disorders such as AD are multi-factorial in origin. Multiple mechanisms may lead to cognitive impairment and involve cellular injury from β-amyloid (Aβ), tau, excitotoxicity, metabotropic receptors, lipid dysfunction, mitochondrial damage, acetylcholine loss, astrocytic cell injury, oxidative stress, heavy metal disease, and cellular metabolic dysfunction with diabetes mellitus (DM) [3,4,8,14,27,35,36,37,38,39,40,41,42,43,44,45,46,47,48,49,50,51,52,53,54]. Present strategies to treat cognitive loss with AD involve therapy with cholinesterase inhibitors that may decrease some symptoms but do not alter disease progression [11,15,52,55]. Additional strategies for dementia can focus on vascular disease [13,14,15,56,57] and metabolic disease, such as DM [3,4,23,26,52,58]. However, additional risk factors for vascular dementia include hypertension, low education in early life, alcohol consumption, and tobacco use that can limit treatment efficacy [3,22,31,59,60,61]. In regard to metabolic disorders, early diagnosis and treatment of DM may offer some degree of protection to inhibit disease progression, but tight serum glucose control cannot completely prevent the complications from DM [5,8,16,62,63,64,65,66,67,68,69,70,71]. In light of the present challenges to overcome cognitive loss, innovative therapeutic strategies are necessary to develop new treatments for dementia. An exciting prospect to effectively target cognitive loss involves the circadian clock gene pathways that involve autophagy, the mechanistic target of rapamycin (mTOR), its associated pathways of mTOR Complex 1 (mTORC1), mTOR Complex 2 (mTORC2), the silent mating type information regulation 2 homolog 1 *(Saccharomyces cerevisiae*) (SIRT1), mammalian forkhead transcription factors (FoxOs), and the trophic factor erythropoietin (EPO) (Table 1).

## 3. Circadian Clock Genes, Neurodegeneration, and Cognitive Loss

Circadian rhythm clock genes play a critical role during neurodegenerative disorders and dementia [8,31,72,73,74,75,76,77,78] (Table 1). In addition, circadian clock genes affect metabolic disease and cell injury [8,78,79,80,81,82,83,84,85,86,87], cell cycle regulation [88,89,90,91], cancer [82,83,92,93,94], energy metabolism and aging [72,76,79,86,95], mitochondrial energy maintenance [78,83,96,97], renal disease [80,92], and viral diseases [74,98,99,100,101,102,103,104,105,106]. The mammalian circadian clock, located in the suprachiasmatic nucleus (SCN) located above the optic chiasm, receives light input from photosensitive ganglion cells in the retina [8,86,103]. The SCN controls melatonin and cortisol release, the temperature of the body, and can respond to oxidative stress [94,107,108]. The basic helix-loop-helix -PAS (Period-Arnt-Single-minded) transcription factor family oversee *Cryptochrome (Cry1 and Cry2)* and *Period (Per1, Per2, and Per3)* genes [8,80,86,92,109,110,111]. CLOCK and BMAL1 [90] are part of the family for clock genes with PER:CRY heterodimers able to block transcription controlled by CLOCK:BMAL1 complexes. CLOCK:BMAL1 complexes also can oversee activity of RORα and NR1D1 (nuclear receptor subfamily 1, group D, member 1), termed retinoic acid-related orphan nuclear receptors REV-ERBα. The REV-ERBα and RORα receptors link up to retinoic acid-related orphan receptor response elements (ROREs). Once present in the BMAL1 promoter, REV-ERBα and ROR can activate and block rhythmic transcription of BMAL1 to lead to circadian oscillation [76,109].

In relation to neurodegeneration and aging with studies involving *Drosophila melanogaster*, lifespan has been observed to be reduced in three arrhythmic mutants involving ClkAR, cyc0 and tim0. ClkAR mutants had significant faster age-related locomotor deficits. Restoring Clk function was able to rescue *Drosophila* from the locomotor deficits. An increase in oxidative stress was noted with the mutant phenotypes, but deficits appeared to correlate best with loss of dopaminergic neurons rather than directly to the presence of oxidative stress in this case [77]. Furthermore, animal models of Parkinson’s disease (PD) with 6-hydroxydopamine (6-OHDA) have shown decreased BMAL1 and RORα persisted with levodopa treatment, suggesting that long-term levodopa treatment may impair circadian rhythm function and potentially lead to cognitive dysfunction [110]. In regard to cognitive impairment, simulated long duration space flight which changes circadian rhythms leads to cognitive decline and potential neuronal injury [112]. In mouse models of AD, changes in the circadian expression of clock gene RNAs have been observed indicating that they may have a role in dementia [113]. The exposure of light altered clock pathway genes in the SCN to include *Cry1*, *Cry2*, and *Per1*. Furthermore, circadian oscillation of BMAL1 has been seen to be changed in AD patients that may lead to impairment in cognition [72].

## 4. Circadian Clock Genes, Neurodegeneration, and Sleep Disruption

Impairments in circadian rhythm that lead to sleep fragmentation can lead to further progression in neurodegenerative disorders. Disruptions in daily activity with frequent international travel, shift work, and artificial lighting can lead to circadian rhythm disturbance [81]. Such observations become more evident when one considers potential inter-planetary travel [114]. These incidences of sleep fragmentation can alter nutritional status and affect vitamin D levels and melatonin release that may progress with oxidative stress and mitochondrial injury [6,83,86]. Interestingly, cerebrospinal fluid is facilitated throughout the brain through a glymphatic pathway that involves a peri-vascular network that is required for the removal of metabolic waste during sleep. This system is dependent upon glial cells and loss of sleep can lead to dysfunction of this system and potential progression of neurodegenerative disorders [115]. Sleep deprivation affects circadian homeostasis and can block the removal of Aβ, tau, α-synuclein that are factors in the pathogenesis of neurodegenerative disorders such as AD and PD. It has been reported that circadian rhythm dysfunction in relation to sleep disturbance can appear in PD patients prior to the onset of motor symptoms [116]. Additionally, loss of a proper sleep-wake cycle with circadian rhythm disruption in elderly individuals has recently been linked to increased risk for COVID-19 infection [74,99,102,104,105,106]. In individuals with DM, sleep fragmentation can worsen metabolic disease especially in patients with obstructive sleep apnea [117] and promote cell injury through oxidative stress [69,118,119,120]. Loss of circadian rhythm with sleep fragmentation also can disrupt organs outside of the nervous system such as the heart and lead to cardiovascular dysfunction [121].

## 5. Circadian Clock Genes and Pathways of Autophagy

Circadian rhythm dysfunction during neuronal injury and cognitive loss has been closely tied to the pathways of autophagy induction [74,83,86,97,119,122,123]. Autophagy has a critical role in multiple neurodegenerative disorders (Table 1). Autophagy can sequester intracellular accumulations that at times may be beneficial during cognitive impairment and AD [14,26,124,125], amyotrophic lateral sclerosis [55,126,127], Huntington’s disease (HD) [14,128], traumatic brain injury [129,130,131], and PD [85,124,129,132,133,134].

Autophagy as part of the programmed death pathways is linked to oxidative stress [5,16,54,73,135,136,137,138,139,140]. Autophagy induction recycles cytoplasmic organelles and components for tissue remodeling [14,141] and can remove non-functional organelles [8,73,136,142]. Macroautophagy recycles organelles in cells and sequesters cytoplasmic proteins into autophagosomes. Autophagosomes subsequently combine with lysosomes to become degraded and start a new course for recycling [14]. Microautophagy leads to lysosomal membrane invagination such that components of the cell cytoplasm are sequestered and digested. Chaperone-mediated autophagy depends upon cytosolic chaperones to move components of the cytoplasm across lysosomal membranes.

Interestingly, changes in the environment that includes sleep loss can decrease memory formation in the hippocampus by affecting autophagy proteins [24,122,143,144,145,146]. At the cellular level, loss of homeostasis [84,119,147] affects circadian rhythm that impairs cognition [14,23,58,86,140,148]. Activation of autophagy with circadian proteins may be protective during stroke. It has been observed that cerebral ischemia is worse if the circadian clock protein PER1 is depressed [123]. In animal models of AD, a circadian control of autophagy is necessary to decrease Aβ accumulation and memory impairment [119,149].

## 6. Circadian Clock Genes and the Mechanistic Target of Rapamycin

Circadian clock gene pathways through autophagy are reliant upon the mechanistic target of rapamycin (mTOR) [8,86,150,151,152] (Table 1). mTOR, a 289-kDa serine/threonine protein kinase, is a critical pathway during neurodegenerative disorders and cognitive loss [3,14,50,58,140,153,154,155]. mTOR is also known as the mammalian target of rapamycin and the FK506-binding protein 12-rapamycin complex-associated protein 1 [14,87,156,157]. mTOR is the primary component of the protein complexes mTOR Complex 1 (mTORC1) and mTOR Complex 2 (mTORC2) [158,159,160]. mTORC1 and mTORC2 are divided into subcomponents [111,140,161,162,163]. mTORC1 has a number of components. These components are the proline rich Akt substrate 40 kDa (PRAS40), mammalian lethal with Sec13 protein 8, termed mLST8 (mLST8), Raptor, and Deptor (DEP domain-containing mTOR interacting protein) [3,25,164]. PRAS40 blocks binding of p70 ribosomal S6 kinase (p70S6K) and the eukaryotic initiation factor 4E (eIF4E)-binding protein 1 (4EBP1) to Raptor that can affect mTORC1 activity [165,166]. Rapamycin mTORC1 [24] as well inhibits activation of mTORC1 [162,167,168,169,170]. mTORC2 has some different components when compared to mTORC1 [171]. These are the protein observed with Rictor-1 (Protor-1), the mammalian stress-activated protein kinase interacting protein (mSIN1), Deptor, mLST8, and Rictor [165,172,173]. mTORC2 can affect migration of cells and changes in the cytoskeleton [174].

Processes that are involved with aging and cognitive loss are dependent upon melatonin, a pineal hormone that controls circadian rhythm [83,94,100], and mTOR in conjunction with autophagy induction [95,175]. Melatonin and the control of the circadian cycle can during aging can be affected infection such as with COVID-19 [99], cellular metabolism [95,108], mitochondrial dysfunction [83], oxidative stress [176,177], and inflammatory mediators [175,178]. Cognitive decline can be associated with the loss of mTOR activity and altered circadian rhythm during prolonged space flight [112]. Cerebral ischemic infarction may be affected by alteration in circadian rhythm genes and fluctuations in mTOR activity [123,150]. In addition, studies suggest that loss of mammalian circadian clock proteins such as period2 (PER2) can lead to enhanced mTOR activity and chemotherapy drug resistance [151].

## 7. Circadian Clock Genes and the Silent Mating Type Information Regulation 2 Homolog 1 *(Saccharomyces cerevisiae*) (SIRT1)

Circadian clock rhythm pathways are critically dependent upon the silent mating type information regulation 2 homolog 1 *(Saccharomyces cerevisiae*) (SIRT1) [8,86,87,91,96,179] (Table 1). SIRT1 is a histone deacetylase that transfers acetyl groups from ε-N-acetyl lysine amino acids to the histones of deoxyribonucleic acid (DNA) to control transcription [14,15,55,86,87,144,180,181,182,183,184,185]. SIRT1 is involved in neurodegenerative disorders [162,184,186,187] that require the modulation of autophagy [120,188,189,190,191,192].

SIRT1 also is closely associated with the mammalian forkhead transcription proteins [24,55,193,194,195,196,197]. The induction of autophagy relies upon mammalian FOXO proteins of the O class that have an important relationship to neurodegenerative disorders [40,162,187,198,199]. For example, central nervous system myelination involving oligodendrocyte progenitor cells is believed to be controlled through FoxO1 transcription factors [200] that may impact disease such as multiple sclerosis [201]. Other work suggests that progressive pathology of demyelinating disorders may be tied to epigenetic changes with DNA methylation and involve genetic variations of FoxO3a and FoxO1 [202]. FoxO activation during autophagy can be beneficial to cell survival, suggesting that a fine balance in FoxO activity may be required to promote cellular protection and survival. For example, FoxOs through the induction of autophagy can lead to the clearance of toxic intracellular accumulations and promote neuronal survival [203,204,205].

In regard to SIRT1, SIRT1 activity leads to increased survival through inhibition of FoxO activity [5,14,194,195,196]. Yet, FoxOs also can bind to the SIRT1 promoter region to alter forkhead transcription [206]. This allows FoxOs to function through autofeedback mechanisms to regulate SIRT1 activity. FoxO proteins, such as those involving FoxO1, have been shown to regulate SIRT1 transcription and increase SIRT1 expression [207]. As a result, it is important to recognize the complex relationship between FoxOs and SIRT1. For example, under some conditions, FoxOs and SIRT1 can function together and synergistically increase the survival of cells. SIRT1 and FoxO3a have been shown to function together to affect cognitive loss and prevent amyloid injury in the brain, mitochondrial dysfunction, and the toxicity of oxidative stress [21,144,208,209].

It is interesting to note that SIRT1 also has an inverse relationship with mTOR [14,189,210,211,212,213]. As previously mentioned, SIRT1 also can significantly affect pathways of autophagy [58,69,161,189,190,191,206,214,215,216]. SIRT1 activity can result in neurite outgrowth and increased neuronal survival during nutrient limiting conditions with the inhibition of mTOR [217]. SIRT1 also can promote tumor cell growth with autophagy activity that requires mTOR inhibition, suggesting that both SIRT1 and autophagy pathways can be targets to control tumor cell growth [215]. SIRT1 is necessary for protection of mitochondrial function in embryonic stem cells during oxidative stress to promote autophagy and inhibit mTOR activity [218]. During periods of hyperglycemia, SIRT1 can offer protection for vascular cells during inhibition of mTOR activity [219]. Blockade of mTOR with SIRT1 activation may also increase cell survival for photoreceptor cells [211] and limit cell senescence [192]. However, some neurodegenerative pathways require a symbiotic relationship between mTOR and SIRT1. For example, under some conditions that may involve dopaminergic neuronal cell loss a balance in activities of SIRT1, mTOR, and FoxOs are required to achieve neuroprotection [220].

Through a number of pathways involving cellular metabolism, the coenzyme ß-nicotinamide adenine dinucleotide (NAD^+^) can play a critical role with circadian rhythm and clock genes that is tied to SIRT1 and mTOR [3,16,74,221,222]. SIRT1 control of circadian rhythm and melatonin may affect cellular glucose tolerance [107], stem cell function [223], and inflammation during obesity [96] and neurodegeneration [178]. In addition, cellular NAD^+^ pools are known to fluctuate with circadian rhythmicity and with aging [74]. If NAD^+^ levels in the cell are diminished, this affects circadian rhythm with cellular levels of nicotinamide to lead to mitochondrial dysfunction and cognitive loss [222]. The circadian rhythm of nicotinamide phosphoribosyl-transferase (NAMPT) is required for NAD^+^ production and is overseen by SIRT1 and the complex of CLOCK:BMAL1. The NAMPT promoter can use SIRT1 to increase production of its own coenzyme [224]. Metformin, an inhibitor of mTOR, can protect SIRT1 activity to maintain proper circadian rhythm of CLOCK and BMAL1 during obesity [221]. Without metformin, the absence of SIRT1 and mTOR block function of CLOCK and BMAL1 during obesity [221]. Yet, other work also suggests that SIRT1 modulates clock genes that involves deacetylation of PER2 [88]. The ability of SIRT1 to oversee several circadian clock gene pathways has suggested that impaired SIRT1 expression can alter circadian rhythm and lead to the loss of cognition and AD [113].

It is of interest to note that SIRT1 regulation of circadian clock genes also may impact cognitive function though trophic factor function, such as erythropoietin (EPO) [159,182,225,226,227]. The *EPO* gene is located on chromosome 7 and is a single copy in a 5.4 kb region of the genomic DNA [228,229]. This gene encodes for a polypeptide chain protein that has initially 193 amino acids [68,230]. EPO is then processed with the removal of a carboxy-terminal arginine^166^ in the mature human and recombinant human EPO (rhEPO). A protein of 165 amino acids with a molecular weight of 30.4 kDa is subsequently generated [231,232,233,234]. EPO expression is present in the brain, uterus, and liver, but the primary site for the production and secretion of EPO is the kidney peritubular interstitial cells [229,230,235,236,237,238]. Expression of EPO is controlled by changes in oxygen tension and not by the concentration of red blood cells [68,239,240].

EPO maintains adipose energy homeostasis in adipocytes to prevent metabolic dysfunction through the combined activation of peroxisome proliferator-activated receptor-α (PPAR-α) and SIRT1 [226]. EPO also fosters cerebral vascular protection through the subcellular trafficking of SIRT1 to the nucleus and prevents mitochondrial depolarization, cytochrome c release, BCL2 associated agonist of cell death (Bad) activity, and caspase activation [225]. EPO enhances survival of human cardiomyocytes through the activation of SIRT1 during chemotherapy toxicity [182]. EPO also blocks the loss of neuronal cells in the brain through the up-regulation of SIRT1 [227]. In addition, EPO can limit cognitive decline during AD [21,29], oversee metabolic pathways [241,242], and prevent mitochondrial dysfunction [7,182,229,243,244,245]. EPO has been shown to increase neuronal survival during toxic environments [246] through pathways that involve PRAS40, mTOR and protein kinase B (Akt) [243,247,248]. EPO relies upon mTOR through autophagy and apoptosis to enhance neuronal survival [3,120,249,250,251,252], foster microglial function [253], and inhibit activity of caspases in the presence of Aβ exposure [254]. Yet, without circadian rhythm oversight, the neuroprotective roles of EPO that involve SIRT1 may not exist. Recent work suggests that clock genes, that include *BMAL1* and *PER2*, are necessary for EPO production, such as during toxic events involving hypoxic insults [255].

## 8. Future Perspectives

**Figure 1:**
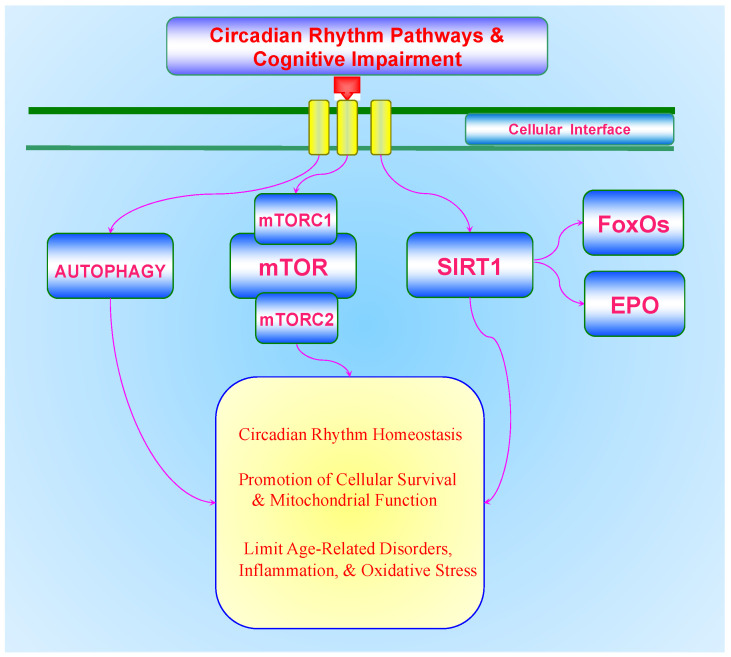
Circadian Rhythm Pathways during Cognitive Impairment. The circadian clock gene pathways provide exciting avenues for the comprehension of the multiple pathways that ultimately can lead to cognitive loss and dementia. These pathways that involve autophagy, the mechanistic target of rapamycin (mTOR), mTOR Complex 1 (mTORC1), mTOR Complex 2 (mTORC2), the silent mating type information regulation 2 homolog 1 *(Saccharomyces cerevisiae*) (SIRT1), mammalian forkhead transcription factors (FoxOs), and erythropoietin (EPO) are complex in nature and require fine biological modulation. Ultimately, circadian rhythm clock genes are critical for the foundation of cognitive health and require maintenance of circadian rhythm homeostasis, prevention of mitochondrial dysfunction that can lead to cellular injury, and preventing the progression of age-related disorders, inflammation, and oxidative stress.

Neurodegenerative disorders are a significant component of NCDs and are increasing in prevalence with the rise in lifespan and advances in healthcare that are occurring throughout the world. With these observations, dementia is now considered to be the 7th leading cause of death throughout the globe and poses a significant financial burden to both developed and developing nations leading to more than 800 billion USD spent to care for individuals with dementia on an annual basis. Furthermore, treatment for cognitive disorders, and especially for diseases such as AD, present enormous challenges since such disorders are multifactorial in origin and current strategies do not offer significant advantages to limit disease progression. As a result, innovative strategies are critical to overcome these challenges. Targeting new therapeutic areas with circadian clock gene pathways that involve autophagy, mTOR, SIRT1, FoxOs and EPO offer new possibilities for the treatment of cognitive impairment (Figure 1).

Alterations in circadian clock genes have been shown to lead to age-related motor deficits, cognitive impairment, and potential onset and progression of AD. Chronic treatment regimens, such as during PD, also may eventually impair circadian rhythm function and affect cognitive loss. At the cellular level, a functional circadian rhythm may be vital to oversee autophagy and prevent cognitive decline and the onset of AD. Additional studies suggest that autophagy induction is necessary to maintain a circadian rhythm homeostasis, such as during chronic sleep fragmentation, and to limit cortical disease that can occur during cerebral ischemia. This fine balance in autophagy activation that may be necessary to maintain proper circadian clock gene regulation is intimately linked to mTOR activity. Loss of mTOR activity can lead to altered circadian rhythm and cognitive loss. Furthermore, fluctuations in the activity of mTOR that can impact autophagy induction can result in increased cerebral ischemia and even chemotherapy drug resistance. Interestingly, SIRT1 has an inverse relationship with mTOR under most circumstances to promote cell survival and mitochondrial function. Through these pathways, SIRT1 can control circadian rhythm to affect aging, cellular metabolic pathways, glucose intolerance, cellular inflammation, cognitive loss, and growth factor neuroprotection with EPO. Interestingly, SIRT1 can regulate the generation of NAD^+^ pools that have been linked to aging during circadian rhythmicity. SIRT1 also is tied to FoxOs such that this complex relationship involves FoxO proteins regulating SIRT1 transcription and increasing SIRT1 expression. As a result, FoxOs and SIRT1 can function synergistically at times to affect cognitive loss and prevent Aβ injury in the brain, mitochondrial dysfunction, and the toxicity of oxidative stress. These observations also correlate with a symbiotic relationship between SIRT1 and mTOR that can occur and involve FoxOs to prevent neurodegenerative cell loss.

## 9. Conclusions

The novel circadian clock gene pathways that involve autophagy, mTOR, and (SIRT1) and include FoxOs and EPO offer exciting prospects for the development of new strategies to understand cognitive loss and to overcome challenges that can limit the onset and progression of dementia (Figure 1). Yet, it is clear that these pathways hold intricate relationships with one another that are dependent upon fine biological controls. Further success of the clinical adaptation of these cellular mechanisms will rest upon elucidating the complex nature of these pathways.

## Figures and Tables

**Table 1:** Highlights—Cognitive Impairment and Dementia: Circadian Clock Gene Pathways.

Neurodegenerative disorders include more than 600 disease entities and currently impact almost one billion individuals throughout the globe, but these numbers are expected to increase with improvements in lifespan and healthcare.
Dementia is the seventh leading cause of death and results in a significant financial burden for all countries throughout the world. Treatment of cognitive disorders is challenging since they are multifactorial and current treatments do not significantly alter disease progression.
Circadian clock gene pathways that involve autophagy, the mechanistic target of rapamycin (mTOR), the silent mating type information regulation 2 homolog 1 *(Saccharomyces cerevisiae*) (SIRT1), mammalian forkhead transcription factors (FoxOs), and erythropoietin (EPO) offer an exciting prospect to target cognitive loss and dementia.
Alterations in circadian rhythm can lead to reduce lifespan, cognitive impairment, behavior abnormalities, and locomotor deficits. Autophagy pathways that oversee circadian rhythm may limit cognitive loss and protect neurons during toxic insults such as ischemia.
Cognitive decline can be associated with the loss of mTOR activity and altered circadian rhythm. Fluctuations of mTOR activity in conjunction with altered circadian rhythm also may lead to cognitive loss as well as neuronal cell death. In contrast, enhanced mTOR activity with loss of PER2 proteins can alter chemotherapy drug efficacy
SIRT1 control of circadian rhythm and melatonin can affect cellular glucose tolerance, inflammation, and cognitive loss. SIRT1 also can regulate the generation of NAD^+^ pools that have been linked to aging during circadian rhythmicity. Although an inverse relationship is usually present, SIRT1 can require mTOR and FoxOs for neuroprotection. SIRT1 also relies upon EPO for energy homeostasis and cellular protection that is based upon intact circadian rhythm function.

## Data Availability

Not applicable.
